# The Combination of Cell Cultured Technology and In Silico Model to Inform the Drug Development

**DOI:** 10.3390/pharmaceutics13050704

**Published:** 2021-05-12

**Authors:** Zhengying Zhou, Jinwei Zhu, Muhan Jiang, Lan Sang, Kun Hao, Hua He

**Affiliations:** 1 Center of Drug Metabolism and Pharmacokinetics, China Pharmaceutical University, Nanjing 210009, China; 3219010221@stu.cpu.edu.cn (Z.Z.); 2020152550@stu.cpu.edu.cn (M.J.); 2 State Key Laboratory of Natural Medicines, Jiangsu Province Key Laboratory of Drug Metabolism and Pharmacokinetics, China Pharmaceutical University, Nanjing 210009, China; 3319071173@stu.cpu.edu.cn (J.Z.); 3120070235@stu.cpu.edu.cn (L.S.)

**Keywords:** in vitro to in vivo translation, human-induced pluripotent stem cells, organoid, microphysiological systems, pharmacokinetic/pharmacodynamic model, quantitative systems pharmacology model, physiologically based pharmacokinetic model

## Abstract

Human-derived in vitro models can provide high-throughput efficacy and toxicity data without a species gap in drug development. Challenges are still encountered regarding the full utilisation of massive data in clinical settings. The lack of translated methods hinders the reliable prediction of clinical outcomes. Therefore, in this study, in silico models were proposed to tackle these obstacles from in vitro to in vivo translation, and the current major cell culture methods were introduced, such as human-induced pluripotent stem cells (hiPSCs), 3D cells, organoids, and microphysiological systems (MPS). Furthermore, the role and applications of several in silico models were summarised, including the physiologically based pharmacokinetic model (PBPK), pharmacokinetic/pharmacodynamic model (PK/PD), quantitative systems pharmacology model (QSP), and virtual clinical trials. These credible translation cases will provide templates for subsequent in vitro to in vivo translation. We believe that synergising high-quality in vitro data with existing models can better guide drug development and clinical use.

## 1. Introduction

Despite the extensive investigation of drug candidates, the attrition rate remains extremely high in drug development [1]. Over 90% of drug candidates entering clinical trials are finally disapproved [2]. Surveys have suggested that more than half of these drug candidates fail in Phases II and III because of a lack of efficacy, and another third of failures are attributed to safety issues [3,4]. Regardless, dismissing ineffective and/or toxic compounds at the early stage of drug development will reduce the cost of failed molecules and prioritise promising candidates [5,6].

**Figure 1:**
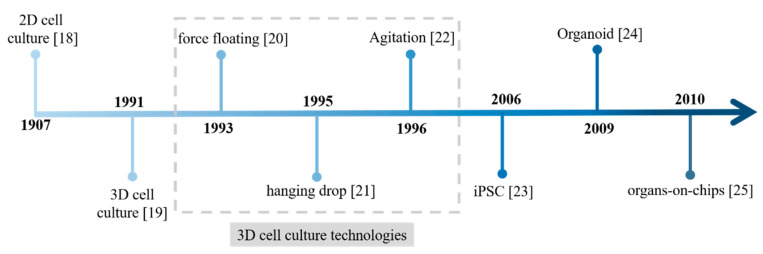
A brief history of the development of in vitro tools. iPSC: induced pluripotent stem cells [18,19,20,21,22,23,24,25].

In drug development, in vitro and animal models are currently essential tools for evaluating the safety and efficacy of a drug candidate before clinical trials [7,8]. They are developed to mimic human disease and then used to predict the response of humans to the drug candidate. Compared to in vitro models, animal models are more acceptable substitutes for human safety and efficacy evaluation, as conventional in vitro models can only be tested in isolation, while animal models exhibit a response after complex in vivo dispositions [9,10]. However, the value of animal models in drug development has long been questioned because of their inaccurate predictability [11]. With the species gap, the biology and mechanisms of a disease are almost impossible to completely mimic by animal models [12,13]. Compared to animal models, cell culture models can be generated with human-derived cells, generating a more human-relevant system to predict the safety and efficacy of a drug candidate [14,15]. Additionally, other advantages of cell culture models include high-throughput compatibility and relative inexpensiveness [16,17]. The recently emerged in vitro models (Figure 1), such as 3D cell culture, organoid, and tissue chips, have been developed to recapitulate the influence of in vivo biology and microenvironmental factors on target cells, performing a more in vivo-relevant response to drug candidates. They are expected to replace animal models in drug development for safety and efficacy testing. However, due to the in vitro–in vivo gap, challenges remain in predicting the safety and efficacy of drug candidates with these advanced cell culture models.

In silico models are effective tools for bridging the preclinical to clinical translational gaps by quantitatively revealing the drug disposition processes and the drug exposure–response relationship [26]. For instance, a physiologically based pharmacokinetic model (PBPK) can integrate our understanding of the anatomy, physiology, physicochemical properties of drug candidates; compound–biological properties; and administration protocol to predict pharmacokinetics (PK) in humans based on preclinical PK studies [27]. Pharmacokinetic/pharmacodynamic models (PK/PD) are extensively utilised in translational pharmacology by quantifying the drug exposure–response relationship [28]. The implementation of quantitative models in drug development has been defined as model-informed drug development (MIDD) and is utilised to integrate preclinical and clinical data sources to facilitate the decision-making process [29]. The application of novel cell culture models will provide an additional data source to address questions in drug development. It emerges as a new challenge to integrate this information and address the questions in drug development for decision-making. Thus, the present review focuses on the application of in silico models from in vitro to in vivo translation.

## 2. Cell Culture Models

**Figure 2:**
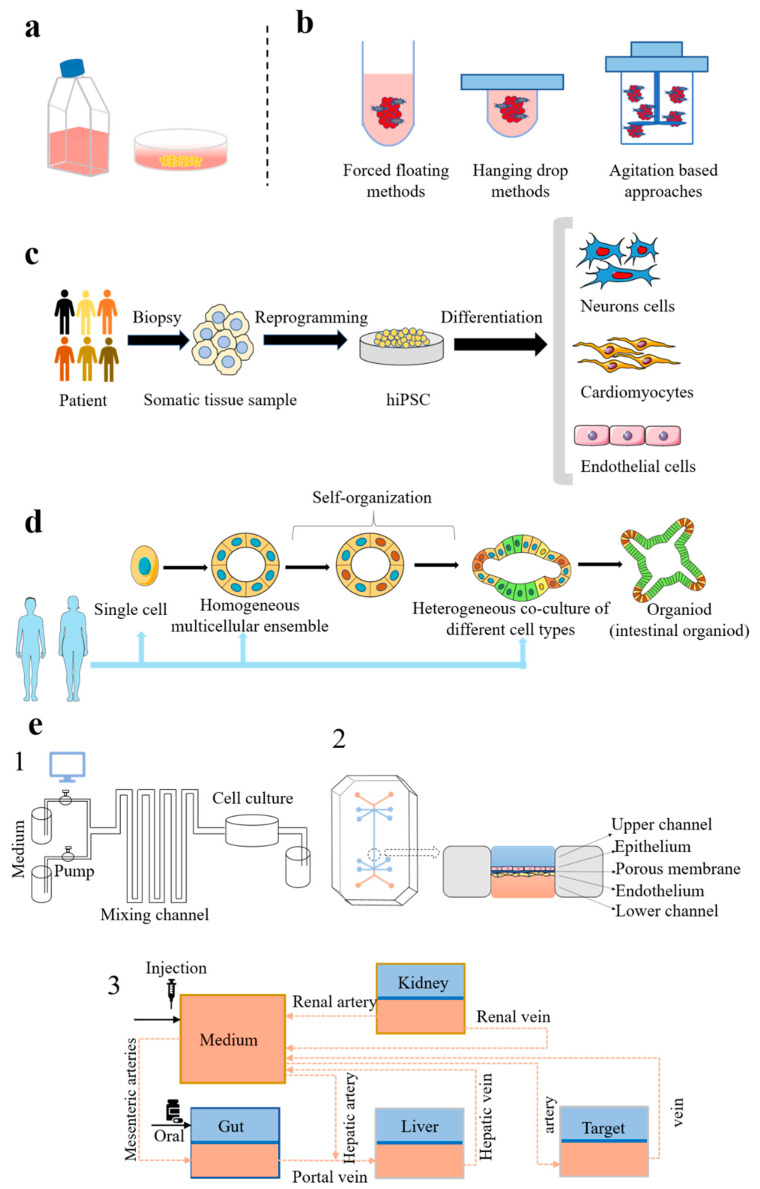
The current various in vitro tools. (**a**) 2D cell culture. (**b**) 3D cell culture. (**c**) hiPSC culture process. Somatic cells are extracted from healthy people or patients and converted into hiPSC through reprogramming. The obtained cells can be differentiated into other cells, such as cardiomyocytes, neurons cells, and endothelial cells. (**d**) Organoid culture process. Organoids can be cultured from human-derived single cell, homogeneous multicellular ensemble, and heterogeneous coculture of different cell types through self-organization. (**e**) Microphysiological systems: (1) controllable and programmable microfluidic cell culture system; (2) structure of organ-on-chips; and (3) integration of or-gan-on-chips, body-on-chips. hiPSC: human-induced pluripotent stem cell.

Cell culture is a rapid and inexpensive method for investigating the disposition, efficacy, and toxicity of drug candidates. Historically, immortalised cell phenotypes or tumour-derived cell lines are cultured on a flat surface to form monolayers of cells, which poorly mimic the complexity of tissues and lack physiological relevance to humans [30]. The recent advances in both cell origins and culture technologies have significantly improved the biological relevance of cell culture models for drug development (Figure 2).

### 2.1. Cell Origins

#### 2.1.1. Primary Cells

Primary cells are harvested or isolated directly from living tissues and usually retain the unique characteristics of the tissue or organ [31]. For example, primary cardiomyocytes preserve contractile function, while primary hepatocytes preserve the metabolic function [32,33]. Primary cells are the most desirable cell tools regarding functionality in drug discovery and development. However, as terminally differentiated cells, their applications are infrequent because of their short lifespan, limited availability, and interdonor variability. For example, the primary cardiomyocytes maintain their beating ability just after they are isolated, and the contractile function will gradually be lost even in an optimum culture environment [34]. For the primary cells with proliferative capacity, they will undergo rapid phenotypic changes. Primary hepatocytes have a strong metabolic capacity in the first generation, but the enzyme content in the cells will decrease or lose after four to five generations [35].

#### 2.1.2. Immortal Cells

Immortal cells are substitute for primary cells. They lose the cell cycle checkpoint pathways and divide indefinitely. They are generated by introducing telomerase or telomerase reverse transcriptase, mutation of cell cycle checkpoints, and transformation of oncogenes and oncoproteins [36]. With the advantages of quick and infinite division and a relatively homogeneous phenotype, immortalised cells provide an inexpensive and stable platform for drug discovery and development. However, the response of immortalised cells to drug candidates might differ significantly from in vivo conditions because of the abnormal gene expression and unreserved functions of tissue or organ. The predictability and in vivo relevance of immortalised cell lines have been greatly doubted [37].

#### 2.1.3. Transfected Cells

Transfected cells are cells with foreign nucleic acids stably or transiently introduced with biological, chemical, or physical approaches to modify the gene expression [38]. The integrated gene can be passed on to offspring in stable transfection, while it cannot be inherited in transient transfection. Virus-mediated biological transfection is currently the most popular approach for transfection with high efficiency and stability. Based on the principle of ‘opposites charges attract’, positively charged nucleic acid/chemical complexes would be attracted to the cell membrane and then deliver the foreign nucleic acids to the cell nucleus in the chemical transfection method. Diverse types of physical transfection methods have been proposed, including micro injection, electroporation, laser-based transfection, and biolistic particle delivery.

In preclinical studies, transfected cell lines expressing specific enzymes, targets, or transporters have been established to determine the interactions between drug candidates and cells. For instance, Madin–Darby canine kidney epithelial cells (MDCK) transfected with the human multidrug resistance (MDR1) gene are recommended to evaluate whether a new drug candidate is a substrate or inhibitor of the transporter P-glycoprotein (P-gp) [39]. Human embryonic kidney cells (HEK293) transfected with human tau441 and mouse neuroblastoma (N2a) cells expressing amyloid precursor protein were implemented to reveal CDT2-controlled cell cycle re-entry in Alzheimer’s disease (AD) [40]. This transfected cell line was developed to screen novel therapeutics for AD.

#### 2.1.4. Induced Pluripotent Stem Cells

In 2006, induced pluripotent stem cells (iPSCs) were successfully constructed by modifying mouse embryos and adult fibroblasts [23]. One year later, they reprogrammed human somatic cells into a pluripotent state [41]. iPSCs are originated from somatic cells and can proliferate and differentiate in vitro. Presently, the successfully differentiated cell lines include endothelial cells, cardiomyocytes, hepatocytes, neuron cells, and mesenchymal cells [42]. Compared to the previously mentioned cell lines, iPSCs have the advantages of humanisation and unlimited division, offering unique opportunities for drug discovery and development, such as disease modelling, drug candidate screening, drug disposition investigation, efficacy, and toxicity evaluation. Nonetheless, the complex reprogramming and differentiating processes remain major challenges for applying iPSCs in drug development. 

With the properties of humanisation and reserved tissue function, iPSCs emerged as potent platforms to screen drug candidates or predict their toxicity. As a rare disease, drug screening for skeletal dysplasias is hindered by the lack of disease model. Yamashita et al. developed degraded cartilage by chondrogenic differentiation of iPSCs generated from patients with thanatophoric dysplasia or achondroplasia. With this disease cell model, stains were identified as drug candidates for skeletal dysplasia therapies. With human-induced pluripotent stem cell-cardiomyocytes (hiPSC-CMs) derived from healthy people or patients, Arun et al. proposed the heart safety index to evaluate drug-induced cardiotoxicity with tyrosine kinase inhibitor (TKI) approved by the US Food and Drug Administration (FDA) [43]. In their study, changes in cardiomyocyte viability, contractility, electrophysiology, calcium processing, and signal transmission were determined after drug exposure, and the results were utilised to calculate the heart safety index. Their results suggested that compounds with ‘heart safety index’ ≤0.1 might possess serious cardiotoxicity. Despite the correct prediction of cardiotoxic drugs, such as vemurafenib and sorafenib, a false positive was also observed. Although the calculated cardiac safety index of imatinib was close to 0.1, no cardiotoxicity was observed in their clinical application. These studies suggest that with the improvement of this technology, hiPSCs could be a potential platform to screen the drug or predict drug-related toxicity for specific tissues or organs.

### 2.2. Cell Origins

#### 2.2.1. 2D Culture

In a 2D cell culture system, cells grow on a flat surface, such as a glass or polystyrene Petri dish. These surfaces provide mechanical support for the cells, and the culture medium supplies the necessary nutrients and growth factors for cell proliferation. The formed 2D monolayer cells can be directly exposed to compounds in culture medium to assay their disposition, efficacy, and/or toxicity on specific cell lines in drug development. Despite the advantages of convenience, reproducibility, and high throughput, a major drawback of a conventional 2D cell culture system is the poor mimic of in vivo conditions because of the differences in cell morphology and expression and the absence of cell–cell and cell–extracellular matrix interactions.

#### 2.2.2. 3D Culture

To bridge the knowledge gap between the cell culture model and real-life in vivo scenarios, a 3D cell culture system has been developed. In this system, cells grow into 3D aggregates or spheroids, which can mimic the cell–cell and cell–extracellular matrix (ECM) interactions and the oxygen and nutrient gradients [44]. Several 3D cell culturing techniques have been developed, such as forced-floating, hanging drop, agitation-based approaches, scaffolds, and matrices [45]. In the forced-floating method, cells are cultured in vessels coated with poly-hydroxyethyl methacrylate (HEMA) or agarose suspensions to promote the formation of a multicellular sphere. In the hanging drop method, the tray with seeded cells is inverted to formulate the hanging drops of the cell suspension under surface tension. In agitation-based approaches, multicellular spheroids are generated by a motional cell suspension with external force under suspension stirring or container rotating. In the scaffold and matrix methods, extracellular supports are provided to support the formation of 3D spheroids. 

Compared to 2D culture, the 3D cell culture system shows a higher relevance on physiopathology and drug response to the in vivo scenario. The overexpressed human epidermal growth factor receptor 2 (HER2) is a major target of breast tumours. SKBR3 cells with overexpressed HER2 were cultured in either 2D or 3D cell culture systems. The HER2 and HER3 heterodimers were formed in a 2D culture, while HER2 homodimers were formed in a 3D culture of SKBR3 cells, resulting in different signalling pathway activation and inconsistent response to trastuzumab, an HER2-targeted antibody. The authors suggested that the 3D culture is more representative of the related signalling pathway in vivo [46]. 

The in vivo drug gradients in tumour tissues can also be mimicked by a 3D cell culture system. In 2D monolayer cells, the drug is accessible to all cells. With the multiple layers of cells in a 3D culture, a limited drug can be delivered to the core of the spheroids, thereby leading to a reduced therapeutic effect. In Tung et al.’s study, 3D cell spheroids and conventional 2D cell monolayers were compared using high-throughput screening instruments [47]. In that study, 5-fluorouracil, a conventional cytotoxic drug, showed a higher antiproliferative effect on a 2D culture compared to a 3D culture. In another two examples, tumour cells grown in a 3D culture system represented a higher resistance to cytotoxic agents, probably because of the lower drug accessibility to tumour cells in the core of the spheroids [48,49].

#### 2.2.3. Organoids

Organoids are referred to as 3D cellular clusters that contain multiple cell types and are capable of self-renewal and self-organisation. Cells in organoids are derived from primary tissue, embryonic stem cells, or induced pluripotent stem cells. A major advantage of organoids is the similarity in organ architecture and functionality between organoids and the original tissue, which has been properly validated by image and animation approaches. Until now, multiple important organs have been mimicked by organoid technology, including the lung, stomach, intestine, liver, kidney, pancreas, prostate, and brain. Organoid biobanks for human cancers, including liver, breast, gastro-intestinal, and bladder cancer, have also been developed [50]. 

As physiologically relevant culture systems, these organoids provide faithful ex vivo models for drug development in a physiologically relevant context. Healthy organoids originating from humans are valuable tools for investigating the PK and toxicity of drug candidates, while disease organoids could be utilised in PD studies. Pharmacokinetically functional intestinal organoids derived from hiPSC have been established to investigate drug absorption in small intestine [51]. Drug disposition-related transporters and metabolic enzymes were successfully expressed in this intestinal organoid, providing a human-relevant tool for PK studies. To screen for hepatotoxic compounds, human liver organoids with bile canaliculi-like architectures were developed using hiPSC [52]. The generated liver organoids were considered a potential assay platform for liver toxicity screening due to their high sensitivity and specificity in predicting the hepatotoxicity of marketed drugs. Organoids derived from colorectal and gastroesophageal cancer patients have been used as preclinical models to predict the clinical responses [53]. The phenotypic and genotypic profiling of these organoids are highly like the original tumours and also the drug response, suggesting the potential values of organoids in the drug candidate screening.

#### 2.2.4. Microphysiological Systems

Organ-on-chips, also known as tissue chips or microphysiological systems (MPS), are microfluidic cell culture devices that recapitulate the functional properties of tissues and organs [54]. The first organ-on-chips were reported by Donald Ingber et al. in 2010 [25]. This lung-on-chips comprised two microchannels separated by a thin, flexible, and porous membrane, which was made from polydimethylsiloxane (PDMS). The flexible membrane was completely covered with ECM, and two types of human-derived lung cells were cultured on the opposite side to simulate alveolar-capillary. The presence of air in the epithelial compartment resulted in the creation of an air–liquid interface to mimic the alveolar. By now, diverse organs-on-chips have been developed to model organs, such as the skin, intestine, liver, kidney, and lung [55,56,57,58,59]. By linking chips mimicked different organs, multiorgan systems have also been established to investigate organ interactions, which provide significant benefits in modelling organ-organ interactions [60].

As innovative devices, there are at least three distinct advantages of organs-on-chips. First, dynamic culture by introducing microfluidic cell culture technology can mimic biomechanical forces in vivo, such as shear stress for blood vessels and stretch forces for lung tissues. Furthermore, the continuous perfusion can also persistently provide nutrients and remove cell metabolites and detritus, which might affect cell growth and differentiation. Second, multiple cell types representing the same or different organs are integrated into chips to mimic a specific organ or the system with multiple organs, providing a new culture system to replicate the organ–organ interactions [61]. Third, the 3D nature and arrangements of the tissues are generated on the platforms, which might promote reproductivity compared to organoids with stochastic self-organised tissue [62]. Nonetheless, significant challenges remain in the development and utilisation of organ-on-chips. Given system complexity, the essentially required components and aspects for the functions of specific organs or tissues should first be identified and then constructed by considering the functional interplay of these components [63,64]. For a multiorgan system, organs related to disease development or drug response should be carefully considered and then integrated. In addition, the culture medium, the cell source and scaffold, and the platform are required to optimise, as the integrated cell types for organ-on-chips require diverse culture conditions.

Regardless of the challenges and complexity, organs-on-chips have emerged as a promising alternative to animal models to address questions in drug development. Traditionally, toxicity assessments involve single-cell type culture, and organoids have advanced it to single tissue or organ culture. In organs-on-chips, multiple organs are integrated to promote the predictability of the physiological relevance of toxicity. For instance, the efficacy and/or toxicity generated by the metabolites of xenobiotics cannot be identified in the absence of metabolic processes. In Chang et al.’s study, microfluidically linked liver-on-a-chip and kidney-on-a-chip were developed to investigate the nephrotoxins and carcinogens of aristolochic acids [65]. In this multiorgan system, the integration of liver-on-a-chip generated human hepatocyte-specific metabolism of aristolochic acid-I, and the kidney-on-a-chip exhibited the cytotoxicity of this metabolite on human kidney proximal tubular epithelial cells, providing a platform to investigate the drug-induced toxicity related to organ–organ interactions. 

PK is determined by the dynamic interactions between blood and drug disposition-related organs, such as the intestine, liver, and kidney. Compared to conventional static mono-cell type culture, organs-on-chips not only provide a dynamic culture system to mimic the blood–organ interactions but also the organ–organ interactions in multiorgan system. Edington et al. established an in vitro PK investigation platform with seven organs to mimic the in vivo disposition of diclofenac, where chips for the liver, gut, lung, heart, pancreas, brain, and endometrium were included and perfused with physiologically related flow rate [66]. The developed multiorgan system could reliably and robustly operate and maintain phenotypic function for three weeks, providing a potential platform for in vitro PK studies.

With human-derived cells to accurately recapitulate their physiopathological properties, organ-on-chips can also be used to evaluate the efficacy of therapeutic agents. Drug efficacy is conventionally assayed under static concentrations in cell culture. Drug exposure magnitude (concentration) and duration (time) are two critical properties associated with drug efficacy. With a dynamic culture system, organ-on-chip technology could mimic the in vivo-like drug concentration, providing a more physiologically relevant platform to determine the efficacy and optimise the therapeutic schedules. In the tumour-on-chip established by Komen et al., a cell culture chamber and a drug-dosing channel are separated by a transparent membrane [67]. Dynamic drug concentration profiles were modelled by controlling the drug concentration in the drug-dosing channel. Compared to static concentration culture, dynamic exposure showed suboptimal efficacy in assaying the antitumour efficacy of oxaliplatin on HCT116 colorectal cancer cells. With in vivo-like drug exposure, organ-on-chip technology could improve the efficacy prediction of in vitro models, facilitating drug candidate screening and development. In addition, the obstacle of the efficacy test of prodrugs by traditional cell culture could also be disrupted using multiorgan chips. For instance, liver-on-chip and tumour-on-chip are integrated to determine the antitumour effect of capecitabine and tegafur, two prodrugs [68].

## 3. Model Informed In Vitro to In Vivo Translation

**Figure 3:**
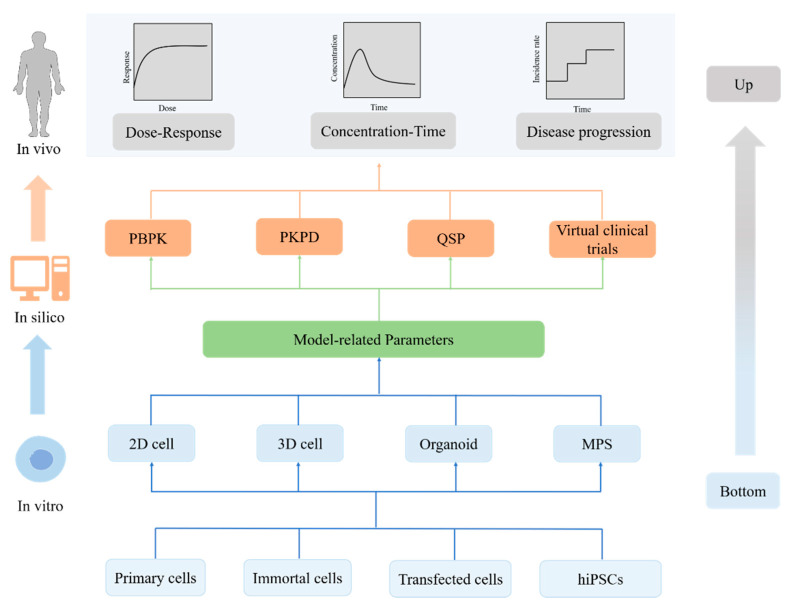
The progress of in vitro to in vivo translation. It is a bottom-up research method. Through high-quality in vitro experimental data and mathematical models, more accurate predictions of in vivo outcome can be obtained. PBPK: physiologically based pharmacokinetic model; PK/PD: pharmacokinetic/pharmacodynamic model; QSP: quantitative systems pharmacology model; MPS: microphysiological system; hiPSC: human-induced pluripotent stem cell.

Despite the innovative and physiological relevance of cell culture models, accurate translation of efficacy and safety from in vitro to in vivo remains a major challenge in drug development (Figure 3). The basic principles of PK, pharmacology, and physiology have long been utilised as the foundation of interspecies translation, and several in silico tools have been established in drug development practice [26]. With an identical clinical PK/PD relationship, these in silico tools can also be promising approaches for in vitro to in vivo translation (Table 1).

### 3.1. Conventional PK/PD Model

PK encompasses the time course of drug concentrations in relevant biological fluids and the effect of intrinsic and extrinsic factors on drug concentrations [69]. PD refers to the relationship between drug exposure and pharmacological or toxicological responses [70]. PK/PD models are mathematical models that quantify the relationship among dose, exposure, and response, providing an alternative to the traditional “dose–response” analysis of drug response [71]. Drug exposure, drug concentration-effect relationship, and physiological turnover and homeostasis are the three essential components in developing PK/PD model. 

In most PK/PD models, plasma drug concentrations, either total or free form, are utilised as the driver of pharmacological and most toxicological effects because of the conveniently blood sampling [72]. Alternatively, tissue concentrations are occasionally accessible and are used to drive the response in PK/PD models [73]. To quantify the drug concentration, compartmental models are usually applied, mostly in plasma and occasionally in hypothetical effective compartments, if a significant delay in response was observed [74]. Drug disposition information from cell culture studies cannot be translated to predict clinical PK, and simple interspecies allometric scaling is more commonly used [75,76]. The mechanism-based PBPK model is a promising approach for the in vitro to in vivo translation of PK, which will be introduced later. 

PD models, such as the sigmoidal E_max_ model, are usually used to establish the concentration-efficacy relationship [77,78]. Capacity of efficacy and potency are PD parameters that link the drug concentration and effect. In the translation of drug efficacy from in vitro to in vivo, inconsistent potency acts as the major obstacle. In most cell culture studies, the target cells are directly exposed to the drug, and the established potency represents the relationship between concentration in cell level and effect. Additionally, the in vivo PD model usually describes the plasma/tissue drug exposure-effect relationship. With the complex drug disposition processes, the cell level’s drug exposure usually differs from plasma/tissue drug exposure, inducing inconsistent potency between in vitro and in vivo conditions [16]. For instance, the tumour static concentration is 27-fold higher in vivo than in vitro studies, according to the in vitro to in vivo correlation analysis of 19 antibody drug conjugates [79]. In this perspective, the cell culture–derived PD model is impossibly directly translated to predict in vivo efficacy. However, considering that the drug distribution is mainly by diffusion, the cell level’s drug exposure is always proportional to plasma/tissue drug exposure and thus the potency. With this consideration, PD models derived from cell culture studies have been directly translated into clinical for proof-of-concept study or indirectly translated to predict clinical efficacy using prior information, such as the clinical efficacy of its same-in-class drugs. 

In our previous studies, direct in vitro to in vivo translation was conducted to address the question of the dosing schedules of chemotherapeutical agents [80]. In clinical practice, the conventional maximum tolerated doses have recently been challenged by the schedule of low doses and high dosing frequency. To address this issue, literature that reported cell culture studies on the antitumour effect of paclitaxel was collected and utilised to develop an in vitro PD model to understand the drug exposure–efficacy relationship. Maximal killing rate (K_max_), drug concentrations yielding 50% of K_max_ (KC_50_), and hill index (γ) were estimated to quantitatively describe the antitumour effect of paclitaxel. The time course of plasma paclitaxel was simulated with a literature-reported PK model and the related model parameters in clinic. In this proof-of-concept study, an approximately rather than exactly clinical response is required to compare the efficacy of paclitaxel under different dosing schedules. The generated PD parameters were then directly integrated into the PK model with an empirical proportion to simulate dosing schedule-dependent efficacy. K_max_in vivo_ was set to a fifth of in vitro value considering tumour growth rate in vivo was comparatively lower than that in vitro system. KC_50_ and γ were integrated directly. This PK/PD model based on simulation suggested consistent results in clinical practice that low doses and high dosing frequency is prior to maximum tolerated doses.

**Table 1:** In Vitro to In Vivo translational model.

In Silico		In Vitro Assay	Examples of In Vitro to In Vivo Translation			
			Technologies Used in Examples	Parameter	In Vitro to In Vivo Translation Result	Application in Drug Development
PBPK	Absorption	Caco-2 [81], MDCK [82], gut MPS [83]	MDCK-MDR1 and Caco-2 [84]	Obtaining half maximal inhibitory concentration (IC_50_) for P-gp and integrating it to models	Demonstrating non-interaction between Axitinib and P-gp substrate	Prediction of drug-drug interaction and exemption of related clinical trials
	Distribution	MDCK [85], hiPSC- brain endothelial cells [86], co-culture [87]	MDCK Ⅱ [88]	Using apparent permeability coefficient (P_app_) to obtain in vitro efflux transporter-mediated clearance and scaling it to the whole-brain in vivo efflux transporter-mediated clearance	Exploring the penetration of AZD1775 across BBB	Prediction of drug distribution and target concentration
	Metabolism	Recombinant enzymes [89], microsomes [90], primary hepatocytes [91], HepG2 [92], HepaRG [93], hESC or hiPSC-hepatocytes [94], liver MPS [95]	Primary hepatocytes [96]	Inputting the intrinsic clearance (CL_int_) to Simcyp software to establish PBPK model	Predicting the difference of AUC in patients with different liver damage after a single oral administration of sirolimus	Prediction of drug metabolism and inter-population extrapolations
	Excretion	MDCK, CHO, HEK-293, HeLa [97], primary cultured renal tubule cells [98], renal MPS [99]	renal MPS [99]	Scaling renal clearance (CL_R_) based on surface area	Predicting human renal excretion for cisplatin and nicotine	Prediction of excretion
PBPK	Integrate ADME	MPS [99]	MPS [99]	Scaling intestinal permeability (Papp) based on absorptive surface, liver clearance (CL_int, in vivo_) based on the number of hepatocytes, renal clearance (CL_R_) based on surface area	Reproducing the clinical PK profiles for both nicotine and cisplatin at different doses and different routes of administration	Simulation of clinical PK profiles
PK/PD		Disease-related cell [100], 2D [80,101], 3D [102], MPS [103], organoids [104]	Six human epithelial cancer cell lines [100]	Directly combining maximal killing rate (K_max_), drug concentrations yielding 50% of K_max_ (KC_50_) and hill index (γ) into in vivo model	Demonstrating that low doses and high dosing frequency for paclitaxel is prior to maximum tolerated doses	Dose and schedule selection
			L540cy cells, Karpas cells [99]	Integrating association and dissociation rate constants (K_on_ and K_off_) to describe the interaction between ADC and target	Predicting therapy in clinical trials employing different dosing regimens	Clinical response prediction
			primary liver cells, red blood cells and brain homogenates [101]	Based on the total enzyme content, scaling metabolic capacity (V_max_) and clearance (CL_int_); Correcting bimolecular inhibition constant (Ki) considering different states of targets in vitro and in vivo	Evaluating the biotoxicity of carbaryl and other carbamates with an anticholinesterase mode of action	Toxicity prediction
			MPS [105]	Based on the number of nephrons in human kidney, scaling maximal injury rate (E_max_) and drug concentrations yielding 50% of E_max_ (EC_50_) into in vivo model	Assessing renal proximal tubule injury caused by three nephrotoxic drugs	Toxicity prediction
QSP (QST)		Disease-related cell [106], 2D [106,107], 3D [107], MPS [108], organoids [109]	Primary hepatocytes [106]	Applying directly the IC50 values for the bile acid transporters to DILIsym, fitting the mitochondrial toxicity parameters (V_max_, K_m_) in MITOsym, and converting them to DILIsym	Explaining the liver toxicity mechanism of PF-04895162 and expound the differences of species	Characterization of target mechanism
			JIMT-1 cells in 2D or 3D and dynamic cell Culture [107]	Integrating drug inhibition or stimulation coefficient (S1p, S2p, Kp etc.) to describe signal pathway molecules perturbation	Optimizing the sequence and inter-dose interval of the three agents (paclitaxel, dasatinib, and everolimus) in the combination	Design of drug administration protocol and evaluation of drug combination
			effector T cells (Teffs), EL4 and E.G7-OVA thymoma cells [110,111,112]	Integrating rate constants defining the half-life of engagement or dissociation between cancer cells and effector T cells (CancerTEng, CancerTInt) directly into the QSP model; scaling number of CD28 receptors expressed on each T cell during priming (CD28_receptors-per-Tcell) by the number of T cell in vivo	Predicting the checkpoint inhibitors’ therapies administered as mono-, combo- and sequential therapies	Clinical response prediction

Abbreviations: Caco-2: human colon adenocarcinoma cells; MDCK: Madin–Darby canine kidney epithelial cells; MPS: microphysiological systems; hiPSC: human-induced pluripotent stem cell; PBPK: physiologically based pharmacokinetic model; PK/PD: pharmacokinetic/pharmacodynamic model; QSP: quantitative systems pharmacology model; QST: quantitative systems toxicology model; hESG: human embryonic stem cell lines; CHO: Chinese hamster ovary cells; HEK-293: human embryonic kidney cells.

Although plasma drug concentrations are always used as the drivers to predict the efficacy, the active drug concentrations at target, especially intracellular target, might be significantly different from plasma [113]. To address this question, Dhaval K Shah et al. utilized in vitro study to clarify the disposition of brentuximab-vedotin in cell level and then developed a cellular PK/PD model to quantify the active drug exposure in L540cy cells and Karpas cells. The generated model parameters, such as association and dissociation rate constants (K_on_, K_off_) between brentuximab-vedotin and CD30, and internalization rate (K_int_) of CD30, were then directly applied to inform the target drug exposure in vivo by integrating the systemic disposition of brentuximab-vedotin. Finally, this PK model was integrated into a PK/PD model to investigate the antitumor effect of brentuximab-vedotin in both tumour-bearing mice and patients [100]. 

Considering the possibility of inconsistent target formations between in vitro and in vivo, translation of PD parameters should be conducted. For instance, acetylcholinesterase (AChE) in the brain and red blood cells usually exists in the form of multimers while it presents as monomer in vitro. The different forms lead to changes of the inhibition constant (K_i_). In Harvey Clewell et al.’s PK/PD model, they translated the in vitro Ki of carbaryl on AChE to in vivo by dividing the fold of monomers in the multimers. For instance, K_iBrain_ is a quarter of K_iin vitro_ because AChE exist in form of tetramer in brain [101].

In clinical practice, drug efficacy is usually evaluated using biomarkers, such as biologically derived indicators. However, the change in biomarkers is determined by both drug effects and pathophysiological processes. In the cell culture system, the growth and metabolism requirements of cells could be completely satisfied by sufficient nutrition in the medium, which differs from the in vivo condition [114]. For instance, the doubling time is around tens of hours for tumour cell culture, and it ranges from tens to thousands of days in clinical practice [115]. Thus, physiological turnover and homeostasis are also critical components in in vitro to in vivo translation. With the empirical approach to describing the physiological turnover and homeostasis in a conventional PK/PD model, in vitro to in vivo translation of these components is usually incorrect [116]. The development of a quantitative systems pharmacology model (QSP) has emerged as a promising approach to solve this problem, which will be introduced later [117].

### 3.2. PBPK Model

As a promising approach for PK simulation, the PBPK model contains arrays of equations with both physiological and drug-related parameters to quantify the absorption, distribution, metabolism, and excretion processes of drug [118]. The physiological parameters, such as the rate of organ blood flows, organ volumes, and rate of lymphatic perfusion, have been well established [119,120]. The drug specific parameters, such as barrier permeability and liver intrinsic clearance, required estimation, and relying on high-quality in vitro and in vivo studies, where cell culture techniques are also included. For instance, human colon adenocarcinoma cells (Caco-2) are usually cultured in transwells to determine intestinal absorption rate [121]. Primary hepatocytes are applied to identify the involved metabolic enzymes and quantify the metabolic rate [122]. A major advantage of the PBPK model is that it integrates drug-specific parameters to predict drug PK. C Emoto et al. obtained the intrinsic clearance (CL_int_) of sirolimus under different CYP450 enzymes by recombinant enzyme incubation experiments. The results were delivered in PBPK model to predict and fit the PK curve of sirolimus. The PBPK model successfully predicted the difference of AUCs in patients with different types of liver damage after a single oral administration [96]. In the study of Nader Sanal et al., a PBPK model based on in vitro experiments was established to predict human brain concentration of AZD1775, a Wee1 tyrosine kinase inhibitor [88]. MDCKII cell monolayers were used to mimic the tight structure of the blood–brain barrier (BBB) and determine the apparent transcellular passive permeability. The active efflux and uptake clearance were estimated based on the results of in vitro interaction experiments, where transfected MDCKII cells and human embryonic kidney (HEK239) cells were implemented. These parameters were integrated into the PBPK model after sensitivity analysis to simulate the PK curve. The prediction of this model has good consistency with the results of a phase 0 clinical trial, where 20 patients took different doses orally before the tumour resection [123]. In this study, in vitro-derived parameters were provided the start point of sensitive analysis and the results suggested a moderate difference (<3-fold) between in vitro and in vivo. With coculture of multiple cell lines to mimic the multilayer structure of BBB, the prediction of active efflux and uptake clearance would be further improved. For instance, Joel W. Blanchard et al. established a 3D cell model consisting of three human-derived cells to mimic the multilayer structure of BBB, which might provide more precise prediction of parameters related to BBB transport [124]. 

With successful in vitro to in vivo translation, the PBPK model has been routinely applied in drug development, including drug–drug interactions (DDIs), clinical PK prediction by interspecies scaling, subpopulation PK prediction by interpopulation scaling, and safety and/or efficacy prediction by target drug exposure prediction [125,126,127,128,129,130]. The occurrence of DDIs seriously affects patient’s safety, thus quantitatively and effectively assessing DDIs is very important [125]. The PBPK model can focus on the main aspects where interactions occur, such as cytochrome P450 (CYP450) enzymes and transporters. According to the current FDA and European Medicines Agency (EMA) guidance documents, the PBPK model is becoming an alternative to clinical trials. Axitinib is an inhibitor of P-gp, which obtained IC_50_ of 3 μM in an in vitro experiment. According to the volume of the gastrointestinal tract, the concentration calculated empirically is 10 times more than the IC_50_, which indicates the risk of potential interaction. However, according to Gastroplus’s ACAT model, drug concentration was found to be 0.0008 μM in the gastrointestinal tract, which is far less than IC_50_. This predicted result provides strong support for the non-interaction between Axitinib and P-gp substrate and promotes the exemption of related clinical trials [84].

Despite an in silico tool, the development of the PBPK model still relies highly on animal studies, as drug disposition in tissues are commonly unclear. The progression of cell culture technologies, such as organoids and MPS, provides new insight into drug disposition in human tissues, which might make animal studies unnecessary with successful in vitro to in vivo translation [131,132]. Herland et al. estimated an outstanding paradigm in human PK prediction with the PBPK model and MPS [133]. They created a first-pass model to mimic drug absorption, metabolism, and excretion with human organ-chips. In this system, human organ chips of the gut, liver, and kidney with endothelium-lined channels were linked to a microfluidic perfusion system with an arteriovenous reservoir. The time course of drug concentrations in the apical and basal channels of organ chips was determined to develop the PBPK model for nicotine. First, model parameters, like liver intrinsic clearance (CL_int_) and renal clearance (CL_R_), were generated by fitting the determined data in individual organ-chip. To get insight into the complex disposition of nicotine in the microfluidic system, the involved models for individual chips were then integrated to validate and optimise the related parameters with experimental results. Finally, physiological parameters in humans were integrated into the model to validate the developed PBPK model by comparing the predicted PK parameters of nicotine, such as C_max_ and half-life, with the reported values. Such an approach performed superior predictability in metabolism properties to conventional interspecies scaling, thereby suggesting its potential to replace animal studies in preclinical PK studies.

Since efficacy and toxicity are driven by drug exposure at the target, the PBPK model could be integrated with the PD model to predict the efficacy and toxicity of drug candidates. In Singh et al.’s study, a PBPK/PD model was developed to facilitate the in vitro to in vivo translation of the chimeric antigen receptor (CAR)-T cell response [134]. In cell culture studies, T cells were cocultured with target cells to determine CAR-T cell expansion with I_max_^CAR-T_Growth^, IC_50_^CAR-T_Growth^, γ^CAR-T_Growth^, CAR-T cell-induced target cell depletion with K^Kill_max^, KC_50_, and cytokine release with K^Cytokine^ and KC_50_^Cytokine^ [135]. A cell-level PD model was first developed to quantitatively describe these results with CAR-T-target complexes as driver. For in vivo translation of these in vitro results, a PBPK model was then developed to describe the PK and distribution of multiple types of CAR-T cells in xenograft mice, including untransducted CAR-T cells, anti-EGFR CAR-T cells, and anti-CD19 CAR-T cells. After validation, the PBPK model was used to predict the CAR-T-target complexes per tumour cell that acted as a linker between the PBPK and PD models. The developed PBPK/PD model was then applied to sensitive analysis to identify the pivotal factor in the antitumour therapy of CAR-T cells.

### 3.3. QSP Model

Cell cultures are pivotal approaches to investigate the biological mechanisms of disease and assay drug–biology interactions [136]. The innovative cell culture technologies provide a more human-relevant platform for drug discovery and development, improving the early assessment of efficacy and toxicity. However, these in vitro data provide limited value for decision making in drug development without successful in vivo translation [137]. The QSP model is an approach to quantitatively analyse dynamic drug–biological system interactions from a systemic perspective to facilitate drug discovery and development [138]. One of the major advantages of the QSP model is the integration of diverse data sources into a system model to address questions in translational pharmacology. With the QSP model as a translational tool, more knowledge will be generated from these novel data types to inform decision making in drug development.

DILIsym^®^, a successful example of QSP/quantitative systems toxicology (QST) application in drug development, was developed to predict drug-induced liver injury [139,140]. It comprises multiple submodels involving the mechanisms of hepatotoxicity, including bile acid transporter inhibition, mitochondrial function alteration, oxidative stress generation, and cholestatic injury to bile duct cells [141]. In DILIsym^®^ development, in vitro toxicity assays are extensively conducted to understand the essential biochemical pathways and estimate the related physiological parameters. For instance, to establish MITOsym, a submodel of DILIsym^®^ for quantifying hepatocellular respiration and bioenergetics, various mitochondrial inhibitors are cocultured with HepG2 cells to generate the model parameters of biochemical processes in mitochondrial bioenergetic responses [142]. Finally, MITOsym was established by integrating all these processes and related parameters with an array of mathematical equations. In practice, the combination of DILIsym^®^ and in vitro toxicity assays have successfully predicted the hepatocyte injury of PF-04895162 that happens solely in humans rather than in rats [106].

In the study by Tanaya R. Vaidya et al., cell culture and the QSP model were combined for therapy optimisation in breast cancer with HER2-targeted therapy resistance [107]. Pathways related to the mechanism of HER2 therapy resistance were integrated using a QSP model. Both a 2D cell culture and a 3D dynamic cell culture of JIMT-1 cells were conducted to investigate the antitumour effect of individual and sequential combination treatment of paclitaxel, dasatinib, and everolimus. Different drug inhibition or stimulation coefficients (e.g., S1p, S2p, Kp etc.) were estimated to describe signal pathway molecules perturbation. These changes in signal pathways finally inhibit the cell growth. A 3D cell culture was conducted in a fluid flow system with drug concentrations varied over time. Different dosing schedules of paclitaxel, dasatinib, and everolimus were tested in these in vitro cell culture systems, with cellular viability and key signalling proteins as effective markers. The QSP-PK/PD model was developed with the observed data and then used to optimise the combined treatment schedule.

In the study of Oleg Milberg et al., a QSP model containing various immune checkpoint receptors, such as cytotoxic T-lymphocyte-associated antigen 4 (CTLA-4), programmed death 1 (PD-1), and programmed death ligand 1 (PD-L1) was developed to assess the checkpoint inhibitors’ therapies administered as mono-, combo-, and sequential therapies [110]. In vitro tools provided parameters to define cellular and molecular species interactions. For instance, rate constants defining the half-life of engagement or dissociation between cancer cells and effector T cells (CancerTEng, CancerTInt) were measured by two-photon microscopy and image analysis [111]. The number of CD28 receptors expressed on each T cell during priming (CD28_receptors-per-Tcell) was obtained by quantitative flow cytometry [112]. CancerTEng and CancerTInt were integrated directly into the QSP model while CD28_receptors-per-Tcell need to be scaled by the number of T cell in vivo. This QSP model was successfully used for combination immunotherapy specific to melanoma.

### 3.4. Virtual Clinical Trials

The combination of innovative cell culture technologies and in silico tools have emerged as promising approaches to improve the prediction accuracy of clinical drug effect. However, challenges remain in the successful in vitro to in vivo translation. In clinical practice, the clinical endpoint rather than biomarker is routinely used to determine efficacy and/or safety [143]. For instance, overall survival and objective response rates are commonly used to evaluate the clinical benefit of antitumour treatment, while tumour growth inhibition is determined in cell culture studies [144,145,146]. To predict the clinical response, it is necessary to translate the graded response in preclinical studies into survival response or frequent response. Additionally, inter-patient variability requires consideration in clinical response prediction [147]. To address these challenges, virtual patients (VPs) were generated to conduct virtual clinical trials [148,149]. VPs are typically defined by an array of pathophysiological parameters, such as gender, age, weight, genotype, phenotype, and biomarkers [150,151]. The values of these parameters can be either simulated with a priori distributions of anthropometric parameters and a particular model or directly resampled from an existing database. To simulate the inter-patient variation, the pathophysiological parameters can be used as covariations of drug-specific parameters in the estimated models, such as PK/PD, PBPK, and QSP models [152,153,154]. With VPs’ estimation, virtual clinical trials can be conducted to predict the clinical response expressed by survival response or frequent response.

In Murat Cirit et al.’s study, a translational PK/PD model based on the renal MPS was established to evaluate drug-induced renal proximal tubule injury [105]. After mimicking clinical drug exposure in the renal MPS, kidney-injury molecule 1 (KIM-1) was monitored to assess the progression of injury. The maximal injury rate (E_max_) and drug concentrations yielding 50% of E_max_ (EC_50_) were estimated to quantitatively describe drug-induced injury. Based on the number of nephrons in human kidney, these parameters were scaled into in vivo PK/PD model. In virtual clinical trials, 100 virtual patients were simulated to assess renal proximal tubule injury caused by three known nephrotoxic drugs (cisplatin, rifampicin, and gentamicin).

In the Phase I clinical trial of pembrolizumab, a monoclonal antibody against PD-1, multiple dosing regimens were identified as safe and effective. To estimate the optimal dosing schedule, a translational PK/PD model was developed based on animal studies and then translated to humans, where PK parameters, such as the slope of drug effect on tumour kill rate (SLtg) and exponent of the power-function of the drug effect (gamma) were derived from clinical PK studies and PD parameters were directly borrowed from the PD model of the xenograft mice model [155]. In virtual clinical trials, a Monte Carlo simulation was conducted with the uncertainty of both PK and PD model parameters and the scaling of tumour growth/shrinkage. Each virtual individual has its own unique time course of drug concentrations and tumour sizes. In clinical practice, tumour size change from baseline was performed as a marker for patient categorisation, including progressive disease, stable disease, intermediate, partial, and major responses. According to such a categorisation, the objective response rate can be estimated to predict the clinical response and dose–response relationship.

## 4. Conclusions

By far, in vitro trial is one of the most important part of preclinical phase. Many examples have been published in which cell-based data have been incorporated into mathematical modelling to accurately and successfully predict in vivo outcomes. However, the current in vitro tools mainly focus on static 2D cultures rather than the novel tools mentioned above. Additionally, these novel tools have not yet been extensively used due to some limitations, such as a high price and complexity. Commercial promotions may reduce high costs. Obviously, to obtain efficacy or toxicity data more rapidly and accurately, the demand for reliable and high-throughput in vitro platforms will continue to increase. It will inspire researchers to make unremitting explorations. 

Currently, drug development depends highly on the in silico model, such as population pharmacokinetic (PopPK), PBPK, PK/PD, and QSP/QST. Regarding drug–drug interactions, PBPK is considered the most promising tool due to its concern for metabolic enzymes and transporters. From preclinical phase to Phase III trials, the PK/PD model maximises therapeutic effects while minimising side effects. Compared with the PK/PD model, the QSP model can better explain the complex mechanisms and processes of disease. However, the existing models need further optimisation and improvement to meet the requirements of in vivo prediction, and the corresponding operation process and specification will be developed. We consider that the QSP model, which integrates physiological models and molecular mechanisms, can better reveal the internal response after administration. Furthermore, synergising the PBPK and QSP models can better demonstrate drug–body interactions.

The in silico model has not only established a bridge between in vitro and in vivo but has also provided an impressive promotion and optimisation for clinical trials. This method enables the selection and optimisation of first-in-human (FIH) and dose regimens, including prediction of human efficacious doses, which improve safety and reduce the attrition rate of clinical trials. Additionally, it can retrospectively analyse the clinical application of the marketed drugs, aiming for a more reasonable dosing regimen optimisation. Currently, many researchers are focusing on in vitro to in vivo translation, for which various in vitro technologies have been developed. Here, we introduced advanced cell culture approaches and in silico models in translation. We hope our review will help reduce drug development costs and improve the translated success rate in the clinical phase.

## Data Availability

Not applicable.
